# The Labyrinth of Renal Cell Carcinoma

**DOI:** 10.3390/cancers12020521

**Published:** 2020-02-24

**Authors:** Claudia Manini, José I. López

**Affiliations:** 1 Department of Pathology, San Giovanni Bosco Hospital, 10154 Turin, Italy; claudiamaninicm@gmail.com; 2 Department of Pathology, Cruces University Hospital, Biocruces-Bizkaia Institute, University of the Basque Country, Plaza de Cruces s/n, 48903 Barakaldo, Bizkaia, Spain

Renal cell carcinoma (RCC) ranks in the top-ten list of malignancies both in males and females [1], and its frequency is increasing as a consequence of the increase in aging and obesity in Western societies [2]. Clear cell renal cell carcinoma (CCRCC) is by far the most common histological variant [3]. CCRCC has received much attention in recent years due to some new therapeutic approaches that are improving the life expectancy of many of these patients. In this way, a tumor traditionally resistant to chemo- and radiotherapy, in which only surgery and early detection had a significant prognostic impact, is becoming ultimately treatable with evident success with antiangiogenic drugs and immune checkpoint blockade, either alone or in combination [4]. However, the problem is far from being solved in many cases, due in part to intra- and inter-tumor heterogeneity [4,5]. 

Roughly 30% of RCCs are other than CCRCC. The list of non-CCRCC tumors grows steadily, and includes classically recognized entities and new ones which are sometimes not yet fully characterized [6]. The maze is particularly complex in the field of RCC with papillary architecture. The classical papillary renal cell carcinoma (PRCC) included types 1 and 2, but today this classification seems insufficient and is no longer recommended [7]. A recent study has identified a new subtype (type 3) with a distinct molecular signature and morphologic overlapping with types 1 and 2 [8]. As a result of this complexity, the diagnosis of PRCC is increasingly becoming a descriptive term among practical pathologists.

To make matters worse, some oncocytic/eosinophilic RCCs (other than ChRCC/oncocytoma) may display papillary, tubule-papillary or solid-papillary architectures. These cases represent a challenge even for experienced pathologists, who used to shelter their diagnoses under the descriptive term “oncocytic papillary renal cell carcinoma”. This descriptive diagnosis, although not very informative, is still valid for the patient since it includes critical data such as tumor grade, necrosis, staging. However, the impression is that the term is too broad for use in daily practice. 

Probably more than in any other human neoplasm, CCRCC and PRCC are hostages of the terminology’s restrictions. Strictly speaking, CCRCC was the classical name given to RCC composed of clear cells and PRCC the one for RCCs architecturally arranged in the papillae, but experience has shown that some CCRCC are not composed of clear cells and some PRCC do not show papillae. Moreover, CCRCC may display a predominantly papillary architecture [9] and PRCC a prominent cytoplasmic clearance [10]. Even worse, some RCC includes different overlapping cell types and architectures, intermingled altogether in different proportions [11]. Currently, we include these cases in the “unclassified” category. The broad spectrum of morphological appearances may be quite confusing, as has been shown in a recent study [12].

Renal oncocytoma (RO) and ChRCC are the best-characterized eosinophilic renal tumors under the microscope [13]. However, a papillary architecture has been very recently described in ChRCC [14], thus favoring diagnostic confusion. The use of the term “oncocytic”, applied to cells with large and deeply granular eosinophilic cytoplasm, is a mistake because, although all oncocytes are eosinophilic, not all eosinophilic cells are oncocytes. As a consequence, the terms eosinophilic and oncocytic are exchanged erroneously with some frequency. The elusive word “hybrid” is applied to those cases that seem to fall in between RO and ChRCC with very unprecise limits [13]. Some of them likely represent genomic RO [15]. Such hybrid oncocytic tumors are also observed in the so-called renal oncocytosis, a condition characterized by multifocal and bilateral oncocytic tumors [16].

Regarding molecular analyses, the issue remains incomplete when considering *VHL* gene malfunctions as the hallmark of CCRCC, and the trisomy of chromosomes 7 and 17 as the signature for PRCC. We know that a subset of CCRCC is *VHL* wild-type [17] and that PRCC may display a wide spectrum of molecular alterations [18]. Therefore, the classification of most RCCs based on molecular signatures is also imperfect and still under construction. Is there any molecular signature specifically linked to the papillary phenotype regardless of the RCC subtype? We do not know the answer to date, but we could hypothesize and, in such a case, the papillary architecture will not be a tumor-specific mark anymore, but a mere trait.

A reductionist prejudice when identifying the varied morphological subtypes of RCC is to link tumor morphology with a precise site of origin along the nephron. As far as we know, a reliable analysis linking CCRCC and PRCC to the proximal convoluted tubule is lacking, and there are no scientific reasons to deny that other elements of the nephron cannot be a potential site of origin for kidney tumors. How might the proximal convoluted tubule be the origin of two different tumors if only one cell type has been histologically described there? This question also remains unanswered, but Gu et al. [19], based on a modeling study on renal cell carcinoma in mice, have proposed that CCRCC may originate in Bowman’s capsule.

The list of new renal cell neoplasms, either recognized as true entities or pending recognition, is still growing, as it has been recently reviewed [6]. Many of them may show some morphologic overlap, so strategic approaches based on immunohistochemistry have been developed trying to overcome this question [20]. The problem at this point is their correct identification in routine practice, since many of them are histologically indistinguishable and are defined only by molecular analyses [21] that are not always performed. This situation leads to the question of how many of these newly described cases are buried in pathology labs under irrecoverable descriptive diagnoses. As several of these diagnoses carry out prognostic and eventually therapeutic implications, the reversal of this situation seems an urgent task for pathologists now that personalized oncology is being increasingly implemented worldwide.

This Special Issue of *Cancers* regards the RCC labyrinth from very different perspectives, including the intimate basic mechanisms governing this disease and the clinical practice principles of their diagnoses and treatments. Thus, the interested reader will have the opportunity to discover some of the most recent findings in renal carcinogenesis and be updated with excellent reviews on new therapeutic approaches and the genetic bases of the disease.

Original articles in this issue show interesting findings with potential clinical application. Examples of the science and research presented in this Special Issue include: the influence of *VHL* deletion in the expression of an unfavorable genetic pattern in CCRCC [22]; how a low dose of curcumin inhibits RCC’s metastatic behavior [23]; the predictive value of the overexpression of EVI1 in CCRCC [24]; the poor outcome of ChRCC patients who lose CDKN1A mRNA and protein expression [25]; the identification of distinct signatures of CCRCC progression through in-depth mapping of urinary *N*-glycoproteome [26]; how a preclinical evaluation method may evaluate the response to targeted therapies in patients with RCC [27]; the RNA sequencing results obtained in two examples of collecting duct renal cell carcinoma, an aggressive rare variant of RCC [28]; how GSTO1*CC genotype predicts shorter survival in CCRCC male patients [29]; the importance of MTA2 as a biomarker of metastatic progression in RCC [30]; the metabolic reprograming in RCC [31]; the prognostic implications of pAMPK immunostaining and its association with SMAD protein expression in CCRCC [32]; the different amount of chromosomal losses in classic ChRCC compared with the eosinophilic subtype of this neoplasm [33]; the potential influence of circular RNAs in CCRCC prognosis [34]; the association of interleukins 4Rα and 13Rα1 with the progression of RCC [35]; the glutathione metabolism in PRCC [36]; how the profiling of primary and metastatic samples of CCRCC reveals a high homology of metastases with a specific subregion of the primary tumor [37]; the interrelationship between serum uric acid levels and RCC survival [38]; and the importance of ghrelin promoting RCC invasion [39]. 

A total of nine reviews have also been published. Predominantly clinical reviews deal with the emerging new therapeutic landscape of metastatic renal cancer [40,41,42,43], the genetic approach to this complex disease [44,45,46,47], and the histopathological diagnostic criteria of newly appearing entities [48].

A brief report shows how hypertonicity-affected genes are differentially expressed in CCRCC correlating with cancer survival [49]. Finally, a short commentary focuses on the therapeutic possibilities of sarcomatoid RCC [50].

## References

[1] Siegel R., Miller K.D., Jemal A. (2020). Cancer statistics, 2020. CA Cancer J. Clin..

[2] Turajlic S., Swanton C., Boshoff C. (2018). Kidney cancer: The next decade. J. Exp. Med..

[3] López J.I. (2013). Renal tumors with clear cells. A review. Pathol. Res. Pract..

[4] Angulo J.C., Lawrie C.H., López J.I. (2019). Sequential treatment of metastatic renal cancer in a complex evolving landscape. Ann. Transl. Med..

[5] Nunes-Xavier C., Angulo J.C., Pulido R., López J.I. (2019). A critical insight into the clinical translation of PD-1/PD-L1 blockade therapying clear cell renal cell carcinoma. Curr. Urol. Rep..

[6] Trpkov K., Hes O. (2019). New and emerging renal entities: A perspective post-WHO 2016 classification. Histopathology.

[7] Akhtar M., Al-Bozom I.A., Al-Hussain T. (2019). Papillary renal cell carcinoma (PRCC): An update. Adv. Anat. Pathol..

[8] Saleeb R.M., Brimo F., Farag M., Rompré-Brodeur A., Rotondo F., Beharry V., Wala S., Plant P., Downes M.R., Pace K. (2017). Toward biological subtyping of papillary renal cell carcinoma with clinical implications through histologic, immunohistochemical, and molecular analysis. Am. J. Surg. Pathol..

[9] Alaghehbandan R., Ulamec M., Martinek P., Pivovarcikova K., Michalova K., Skenderi F., Hora M., Michal M., Hes O. (2019). Papillary pattern in clear cell renal cell carcinoma: Clinicopathologic, morphologic, immunohistochemical and molecular genetic analysis of 23 cases. Ann. Diagn. Pathol..

[10] Klatte T., Said J.W., Seligson D.B., Rao P.N., de Martino M., Shuch B., Zomorodian N., Kabbinavar F.F., Belldegrun A.S., Pantuck A.J. (2011). Pathological, immunohistochemical and cytogenetic features of papillary renal cell carcinoma with clear cell features. J. Urol..

[11] Michalova K., Steiner P., Alaghehbandan R., Trpkov K., Martinek P., Grossmann P., Montiel D.P., Sperga M., Straka L., Prochazkova K. (2018). Papillary renal cell carcinoma with cytologic and molecular genetic features overlapping with renal cell oncocytoma: Analysis of 10 cases. Ann. Diagn. Pathol..

[12] Cai Q., Christie A., Rajaram S., Zhou Q., Araj E., Chintalapati S., Cadeddu J., Margulis V., Pedrosa I., Rakheja D. (2019). Ontological analyses reveal clinically-significant clear cell renal cell carcinoma subtypes with convergent evolutionary trajectories into an aggressive type. EBioMedicine.

[13] Williamson S.R., Gadde R., Trpkov K., Hirsch M.S., Srigley J.R., Reuter V.E., Cheng L., Kunju L.P., Barod R., Rogers C.G. (2017). Diagnostic criteria for oncocytic renal neoplasms: A survey of urologic pathologists. Hum. Pathol..

[14] Michalova K., Tretiakova M., Pivovarcikova K., Alaghehbandan R., Montiel D.P., Ulamec M., Osunkoya A., Trpkov K., Yuan G., Grossmann P. (2020). Expanding the morphologic spectrum of chromophobe renal cell carcinoma: A study of 8 cases with papillary architecture. Ann. Diagn. Pathol..

[15] Poté N., Vieillefond A., Couturier J., Arrufat S., Metzger I., Delongchamps N.B., Camparo P., Mège-Lechevallier F., Molinié V., Sibony M. (2013). Hybrid oncocytic/chromophobe renal cell tumors do not display genomic features of chromophobe renal cell carcinomas. Virchows. Arch..

[16] Giunchi F., Fiorentino M., Vagnoni V., Capizzi E., Bertolo R., Porpiglia F., Vatrano S., Tamberi S., Schiavina R., Papotti M. (2016). Renal oncocytosis: A clinicopathological and cytogenetic study of 42 tumours occurring in 11 patients. Pathology.

[17] Turajlic S., Xu H., Litchfield K., Rowan A., Horswell S., Chambers T., O’Brien T., Lopez J.I., Watkins T.B., Nicol D. (2018). Deterministic evolutionary trajectories influence primary tumor growth: TRACERx *Renal*. Cell.

[18] Cancer Genome Atlas Research Network (2016). Comprehensive molecular characterization of papillary renal cell carcinoma. N. Eng. J. Med..

[19] Gu Y.-F., Cohn S., Christie A., McKenzie T., Wolff N., Do Q.N., Madhuranthakam A.J., Pedrosa I., Wang T., Dey A. (2017). Modeling renal cell carcinoma in mice: *Bap1* and *Pbrm1* inactivation drive tumor grade. Cancer Discov..

[20] Kryvenko O.N., Jorda M., Argani P., Epstein J.I. (2014). Diagnostic approach to eosinophilic renal neoplasms. Arch. Pathol. Lab. Med..

[21] Beaumont M., Dugay F., Kammerer-Jacquet S.-F., Jaillard S., Cabillic F., Mathieu R., Verhoest G., Bensalah K., Rioux-Leclercq N., Belaud-Rotureau M.-A. (2019). Diagnosis of uncommon renal epithelial neoplasms: Performances of fluorescence in situ hybridization. Hum. Pathol..

[22] Groß A., Chernyakov D., Gallwitz L., Bornkessel N., Edemir B. (2020). Deletion of Von Hippel–Lindau Interferes with Hyper Osmolality Induced Gene Expression and Induces an Unfavorable Gene Expression Pattern. Cancers.

[23] Rutz J., Maxeiner S., Justin S., Bachmeier B., Bernd A., Kippenberger S., Zöller N., Chun F., Blaheta R. (2020). Low Dosed Curcumin Combined with Visible Light Exposure Inhibits Renal Cell Carcinoma Metastatic Behavior In Vitro. Cancers.

[24] Palomero L., Bodnar L., Mateo F., Herranz-Ors C., Espín R., García-Varelo M., Jesiotr M., Ruiz de Garibay G., Casanovas O., López J. (2020). EVI1 as a Prognostic and Predictive Biomarker of Clear Cell Renal Cell Carcinoma. Cancers.

[25] Ohashi R., Angori S., Batavia A.A., Rupp N.J., Ajioka Y., Schraml P., Moch H. (2020). Loss of CDKN1A mRNA and protein expression are independent predictors of poor outcome in chromophobe renal cell carcinoma patients. Cancers.

[26] Santorelli L., Capitoli G., Chinello C., Piga I., Clerici F., Denti V., Smith A., Grasso A., Raimondo F., Grasso M. (2020). In-depth mapping of the urinary *N*-glycoproteome: Distinct signatures of ccRCC-related progression. Cancers.

[27] Roelants C., Pillet C., Franquet Q., Sarrazin C., Peilleron N., Giacosa S., Guyon L., Fontanell A., Fiard G., Long J.-A. (2020). Ex-vivo treatment of tumor tissue slices as a predictive preclinical method to evaluate targeted therapies for patients with renal carcinoma. Cancers.

[28] Wach S., Taubert H., Weigelt K., Hase N., Köhn M., Misiak D., Hüttelmaier S., Stöhr C.G., Kahlmeyer A., Haller F. (2020). RNA sequencing of collecting duct renal cell carcinoma suggests an interaction between miRNA and target genes and a predominance of deregulated solute carrier genes. Cancers.

[29] Radic T., Coric V., Bukumiric Z., Pljesa-Ercegovac M., Djukic T., Avramovic N., Matic M., Mihailovic S., Dragicevic D., Dzamic Z. (2019). *GSTO1*CC* genotype (rs4925) predicts shorter survival in clear cell renal cell carcinoma male patients. Cancers.

[30] Chen Y.-S., Hung T.-W., Su S.-C., Lin C.-L., Yang S.-F., Lee C.-C., Yeh C.-F., Hsieh Y.-H., Tsai J.-P. (2019). MTA2 as a potential biomarker and its involvement in metastatic progression of human renal cancer by miR-133b targeting MMP-9. Cancers.

[31] Bogusławska J., Popławski P., Alseekh S., Koblowska M., Iwanicka-Nowicka R., Rybicka B., Kędzierska H., Głuchowska K., Hanusek K., Tański Z. (2019). MicroRNA-mediated metabolic reprograming in renal cancer. Cancers.

[32] Jung M., Lee J.H., Lee C., Park J.H., Park Y.R., Moon K.C. (2019). Prognostic implication of pAMPK immunohistochemical staining by subcellular location and its association with SMAD protein expression in clear cell renal cell carcinoma. Cancers.

[33] Ohashi R., Schraml P., Angori S., Batavia A.A., Rupp N.J., Ohe C., Otsuki Y., Kawasaki T., Kobayashi H., Kobayashi K. (2019). Classic chromophobe renal cell carcinoma incur a larger number of chromosomal losses than seen in the eosinophilic subtype. Cancers.

[34] Franz A., Ralla B., Weickmann S., Jung M., Rochow H., Stephan C., Erbersdobler A., Kilic E., Fendler A., Jung K. (2019). Circular RNAs in clear cell renal cell carcinoma: Their microarray-based identification, analytical validation, and potential use in a clinico-genomic model to improve prognostic accuracy. Cancers.

[35] Kang M., Lee J., Ha S., Lee C., Kim K., Jang K., Park S. (2019). Interleukin4Rα (IL4Rα) and IL13Rα1 Are Associated with the Progress of Renal Cell Carcinoma through Janus Kinase 2 (JAK2)/Forkhead Box O3 (FOXO3) Pathways. Cancers.

[36] Ahmad A., Paffrath V., Clima R., Busch J., Rabien A., Kilic E., Villegas S., Timmermann B., Attimonelli M., Jung K. (2019). Papillary Renal Cell Carcinomas Rewire Glutathione Metabolism and Are Deficient in Both Anabolic Glucose Synthesis and Oxidative Phosphorylation. Cancers.

[37] Ferronika P., Hof J., Kats-Ugurlu G., Sijmons R.H., Terpstra M.M., de Lange K., Leliveld-Kors A., Westers H., Kok K. (2019). Comprehensive profiling of primary and metastatic ccRCC reveals a high homology of the metastases to a subregion of the primary tumour. Cancers.

[38] Yim K., Bindayi A., McKay R., Mehrazin R., Raheem O., Field C., Bloch A., Wake R., Ryan S., Patterson A. (2019). Rising Serum Uric Acid Level Is Negatively Associated with Survival in Renal Cell Carcinoma. Cancers.

[39] Lin T., Yeh Y., Fan W., Chang Y., Lin W., Yang T., Hsiao M. (2019). Ghrelin Upregulates Oncogenic Aurora A to Promote Renal Cell Carcinoma Invasion. Cancers.

[40] Terren I., Orrantia A., Mikelez-Alonso I., Vitallé J., Zenarruzabeitia O., Borrego F. (2020). NK cell-based immunotherapy in renal cell carcinoma. Cancers.

[41] Garje R., An J., Greco A., Vaddepally R.K., Zakharia Y. (2020). The future of immunotherapy-based combination therapy in metastatic renal cell carcinoma. Cancers.

[42] Brighi N., Farolfi A., Conteduca V., Gurioli G., Gargiulo S., Gallà V., Schepisi G., Lolli C., Casadei C., De Giorgi U. (2019). The interplay between inflammation, anti-angiogenic agents, and immune checkpoint inhibitors: Perspectives for renal cancer treatment. Cancers.

[43] Angulo J.C., Shapiro O. (2019). The changing therapeutic landscape of metastatic renal cancer. Cancers.

[44] Caliò A., Segala D., Munari E., Brunelli M., Martignoni G. (2019). MiT family translocation renal cell carcinoma: From early descriptions to the current knowledge. Cancers.

[45] Alaghehbandan R., Perez Montiel D., Luis A.S., Hes O. (2020). Molecular genetics of renal cell tumors: A practical diagnostic approach. Cancers.

[46] Carril-Ajuria L., Santos M., Roldán-Romero J.M., Rodriguez-Antona C., de Velasco G. (2020). Prognostic and predictive value of *PBRM1* in clear cell renal cell carcinoma. Cancers.

[47] Debien V., Thouvenin J., Lindner V., Barthélémy P., Lang H., Flippot R., Malouf G. (2020). Sarcomatoid Dedifferentiation in Renal Cell Carcinoma: From Novel Molecular Insights to New Clinical Opportunities. Cancers.

[48] Siadat F., Trpkov K. (2020). ESC, ALK, HOT, LOT: Three letter acronyms of emerging renal entities knocking on the door of the WHO classification. Cancers.

[49] Kandabarau S., Leiz J., Krohn K., Winter S., Bedke J., Schwab M., Schaeffeler E., Edemir B. (2020). Hypertonicity-Affected Genes Are Differentially Expressed in Clear Cell Renal Cell Carcinoma and Correlate with Cancer-Specific Survival. Cancers.

[50] Pichler R., Compérat E., Klatte T., Pichler M., Loidl W., Lusuardi L., Schmidinger M. (2019). Renal Cell Carcinoma with Sarcomatoid Features: Finally New Therapeutic Hope?. Cancers.

